# Demography of vascular Behcet’s disease with different gender and age: an investigation with 166 Chinese patients

**DOI:** 10.1186/s13023-019-1061-1

**Published:** 2019-04-29

**Authors:** Yong Chen, Jian-Fei Cai, Chen-Hong Lin, Jian-Long Guan

**Affiliations:** 1 Rheumatology and Immunology Department of Huadong Hospital Affiliated to Fudan University, #221 yan’an west Road, Jingan District, Shanghai, 200040 People’s Republic of China; 2 Present address: Department of Rheumatology and Immunology, Hospital of Integrated Traditional Chinese and Western Medicine, Southern Medical University, Guangzhou, 510330 China

**Keywords:** Behcet’s disease, Thrombosis, Aneurysm, Epidemiology

## Abstract

**Background:**

The Clinical features of vascular Behcet’s disease (BD) are not well understood because there are few studies. Our study aimed to investigate characteristics of vascular BD in both genders in different age groups.

**Results:**

We enrolled 923 patients with BD who presented to our hospital with adequate medical histories and proper vascular screening exams. The raw incidence rate of vascular BD was 17.98% (166/923). The ratio of vascular BD in male to female patients was 1.868 (*p* = 0.0004, 95% confidence interval (CI): 1.317 to 2.625). There was a tendency towards higher ESR and CRP in vascular BD patients than in mucocutaneous, but the difference was not significant. The most susceptible affected vessels were cerebral (29.6% in males, 59.4% in females) and lower limb vessels (31.2% in males, and 17.2% in females). The incidence of vascular involvement in younger (< 50 years old) and older (≥ 50 years old) patients were similar, with ratios of 16.58% (122/736) and 23.53% (44/187) respectively. However, in females, younger patients were less likely to have vascular involvement than were older patients (11.43% vs. 20% *p* = 0.0328, OR: 0.5161, 95% CI: 0.2874 to 0.912). Aneurysm or pseudoaneurysm was diagnosed in 1.84% (17/923) patients, mostly in male patients (*p* < 0.05, OR: 3.221, 95% CI: 1.097 to 9.112). Twenty vascular BD patients were followed up, and the age at BD diagnosis was 33.23 ± 11.56 year. This did not differ statistically with their age at vascular involvement (36.15 ± 9.52 years). Ages of vascular BD patients did not differ significantly from those of mucocutaneous BD patients (*n* = 143) in both males and females.

**Conclusion:**

Vascular BD, including lethal types of aneurysm is more likely to occur in male patients. The female patients has a similar incidence rate with the males in their postmenopausal age. There was no evidence of progression course from mucocutaneous BD to vascular involvement.

## Background

Behcet’s disease (BD) is a vasculitis, manifesting as a chronic, relapsing autoinflammatory disorder. It is characterized by oral and genital ulcerations with uveitis, as well as other clinical manifestations in multiple organ systems [1]. BD is also known as Silk Road disease, with a 2000 year history in China, the location of one of the most susceptible populations [2]. The incidence of vascular involvement in BD is reported to be in the range of 5–30% [3]. Vascular BD is divided into three subtypes: venous occlusions, arterial occlusions, and arterial aneurysms. Arterial lesions pose the greatest risks. The most common arterial lesions are occlusions, stenoses and aneurysms or pseudoaneurysms. Therefore, vascular complications in BD patients are regarded as a life-threatening situations [4]. The cause of vascular BD is not well-understood, but it is primarily characterized by auto-inflammation of the blood vessels. Therefore, the objective of treatment in vascular BD is suppression of inflammation or autoimmunity, with prevention of complications. The role of anticoagulants is controversial in the management of BD associated with thrombophlebitis [5].

Because BD is a rare disorder and BD with vascular involvement is regarded as an orphan disease. Apart from some case reports, there are few clinical studies of vascular BD published. Lacking sufficient knowledge regarding vascular BD limits our understanding to diagnose and manage this manifestation. To better understand vascular BD, we retrospectively investigated 166 cases of vascular BD in 923 BD patients from China.

## Results

### General vascular BD incidence in BD patients

**Fig. 1:**
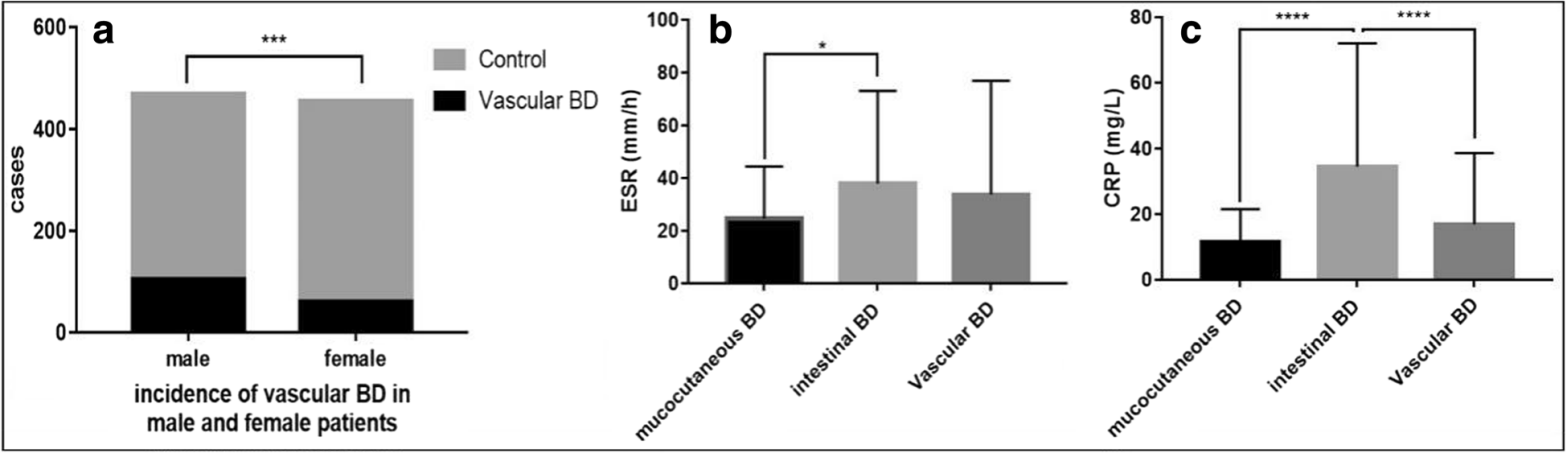
**a** Male patients were more susceptible than females to vascular involvement (OR: 1.868, p = 0.0004, 95% CI: 1.317 to 2.625. Control: BD patients without vascular involvement). **b** and **c** Vascular BD patients had higher ESR and CRP than did mucocutaneous BD patients (without statistical significance). CRP was lower than that of intestinal BD patients (*p* < 0.0001)

In total, 166/923 (17.98%) patients had vascular BD. The mean age of these 166 vascular BD patients were 40.05 years (range 13 to 74). There were 105 men (63.25%) and 61 women (36.75%). The 923 BD patients included 468 men (50.7%) and 455 women (49.3%). Fisher’s exact test indicated odds ratio (OR) for vascular BD in male to female patients was 1.868 (*p* = 0.0004, 95% CI: 1.317 to 2.625) (Fig. 1a).

We measured ESR and CRP in BD patients with only mucocutaneous involvement (*n* = 143), and those with intestinal BD (*n* = 56) and compared values with those of vascular BD patients. Vascular BD patients had higher ESR (33.76 ± 43.14 mm/h) than did mucocutaneous BD patients (24.61 ± 19.81 mm/h), but without statistical significance. Intestinal BD (37.98 ± 34.97 mm/h) patients had higher ESRs than did mucocutaneous BD patients (*p* < 0.05, Fig. 1b) and similar values as vascular BD patients. However, the CRP in vascular BD patients (16.87 ± 21.83 mg/L) was not significantly different from that of mucocutaneous BD patients (11.67 ± 9.81 mg/L), and was lower than that of intestinal BD patients (34.51 ± 37.55 mg/L, *p* < 0.0001) (Fig. 1c).

### Body distribution of vascular involvement

**Fig. 2:**
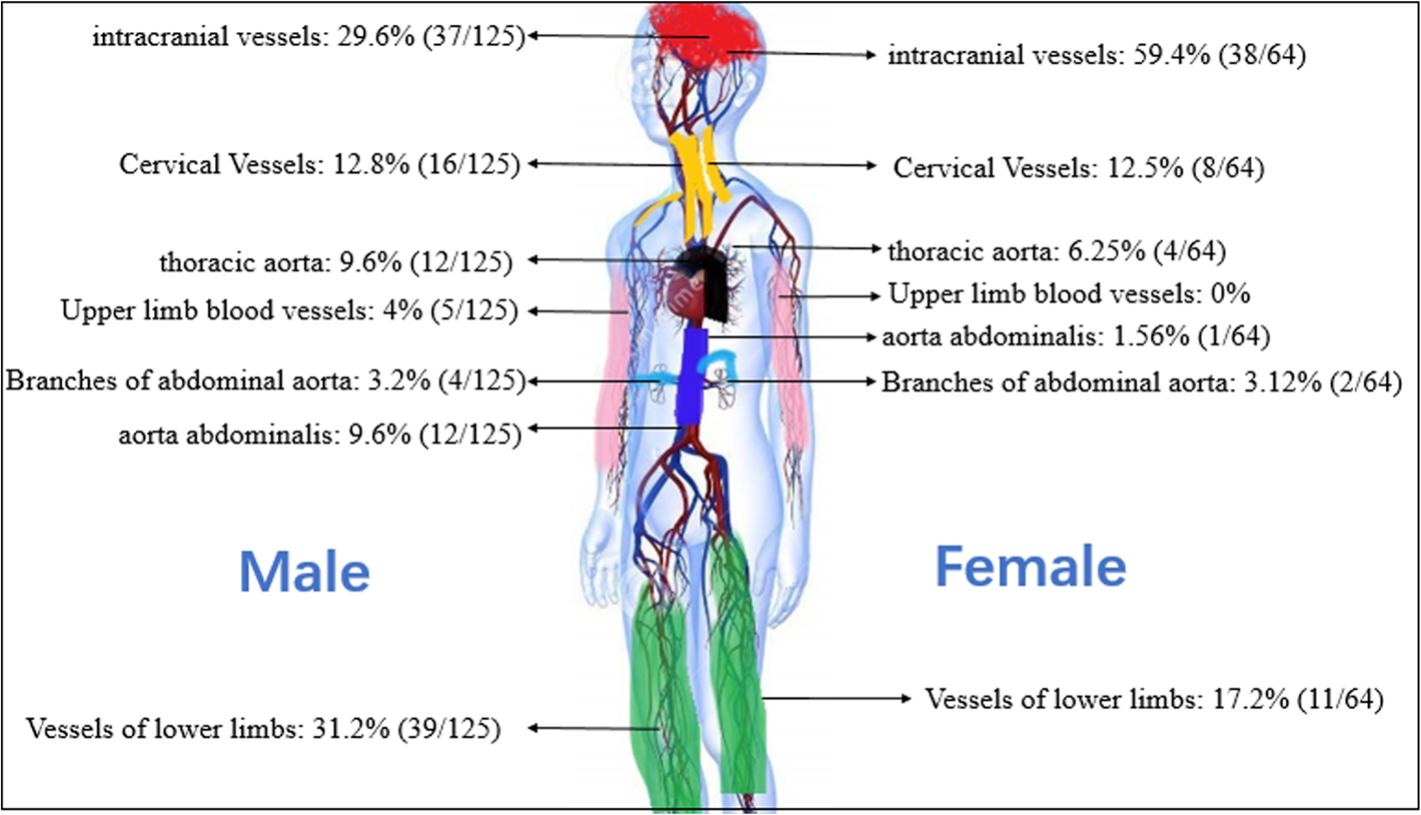
Involved vessels or organs in men and women (denominator represents the quantity of organs or vessels involved in men and women)

15/105 (14.3%) male and 3/61 (4.9%) female patients had more than one [2, 3] vessels involved. In male patients, there were 125 vessels involved, versus 64 in women. The most susceptible organ was brain, with 29.6% (37/125) and 59.4% (38/64) affected vessels in the male and female groups respectively. The intracranial vessels usually manifested as multiple ischemic foci. The percentages of affected vessels according to the location in the male and female patients are showed in Fig. 2.

One (1.6%) female patient suffered an aneurysm in the distal basilar artery (diameter of 8 mm). Sixteen (15.2%) males and 8 (13.1%) female patients had neck vessels involvement, including the common carotid artery, usually manifesting as endarterial hypertrophy or atherosclerotic plaque. One (1.6%) female patient had aneurysmal dilatation of the jugular vein. Five (4.8%) male patients showed thromboses in upper limbs vessels including the median cubital and brachiocephalic veins in. Twelve (11.4%) males and 4 (6.6%) females had involvement of the aorta or its branches. The most common pathological change was aortic aneurysm or pseudoaneurysm. Others included the precaval region, the innominate artery; and 1 (1%) male patient manifested with coronary heart disease. Ten (9.5%) males and a female (1.6%) manifested as abdominal aorta involvement, 2 of the male patients presented with ulceration of the vessel, the other males were diagnosed with aneurysm or pseudoaneurysms. Eight (7.6%) males and 2 (3.3%) females had involvement of branches of the abdominal aorta, including thrombosis or stenosis of the celiac trunk, superior mesenteric artery or renal artery. Thirty nine (37.1%) males and 11 (18.0%) females had lower limbs vessels involvement. These included thrombosis of the femoral vein, great saphenous vein, deep vein, and atherosclerosis of common femoral artery, superficial femoral artery, deep femoral artery and popliteal artery.

### Relationship between vascular involvement and age

**Fig. 3:**
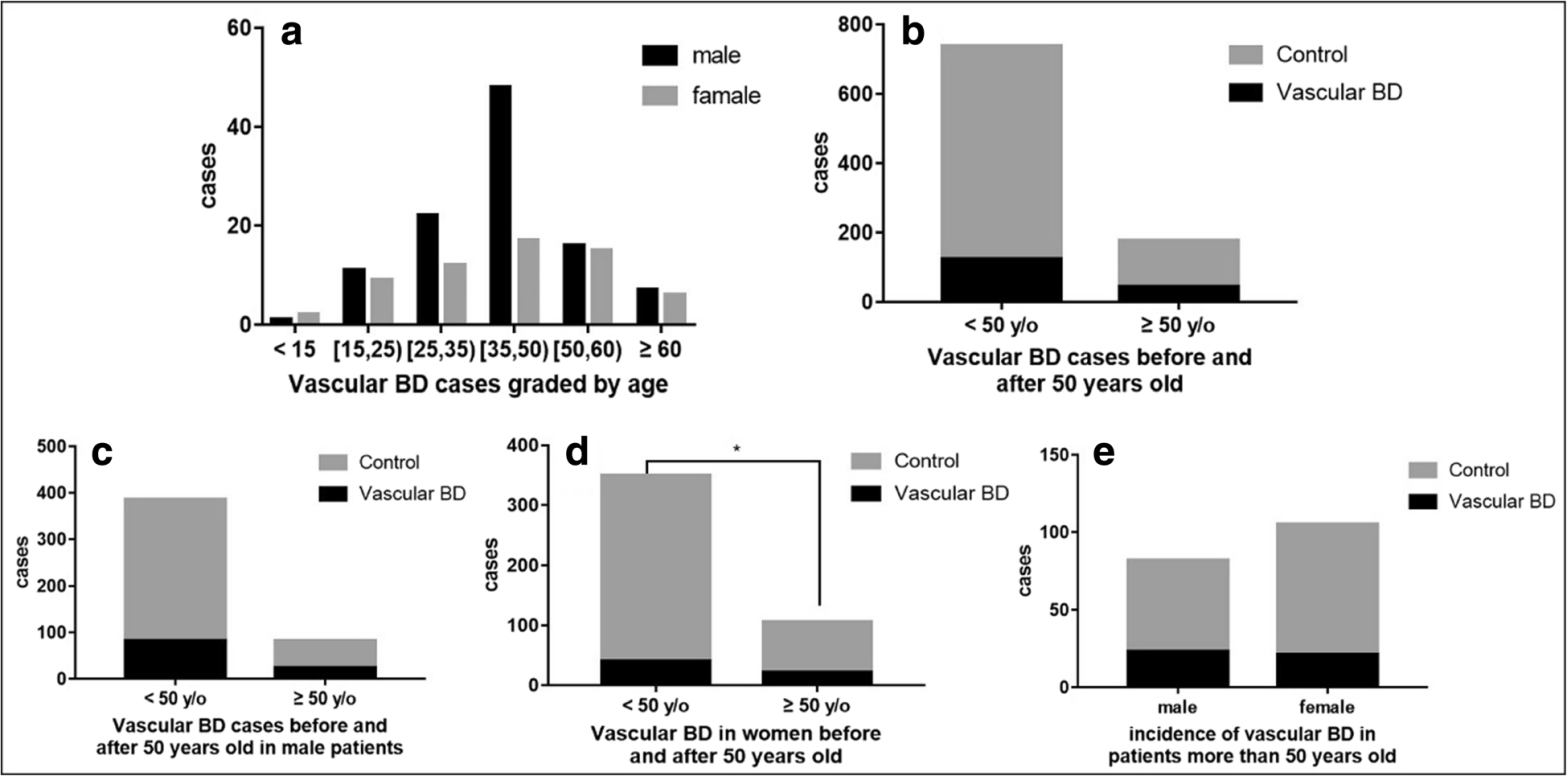
Younger female BD patients were less likely to develop vascular involvement (*p* = 0.0328, OR: 0.5161, 95% CI: 0.2874 to 0.912). However, after menopause, the incidence was similar to that of male patients. **a** Black and gray bars represent male and female patients respectively. **b**-**e** Black bars represent BD patients with vascular involvement while gray bars represent BD patients without vascular involvement

All ages of BD patients could presented with vascular involvement (Fig. 3a). Of all 166 vascular patients, 73.49% (122/166) were less than 50 years old and 26.51% (44/166) were equal to or greater than 50 years old. The incidence of vascular involvement in younger or older patients were similar, with ratios of 16.58% (122/736) and 23.53% (44/187) respectively (Fig. 3b). In male patients only, the incidence of vascular involvement in the young or the elderly were without statistical significance, with percentages of 17.52% (82/468) and 28.05% (23/82) (Fig. 3c).

The situation was different in female patients. The vascular involvement in BD patients younger than 50 years old (11.43%, 40/350) was less than that of older patients (20%, 21/105) (*p* = 0.0328, OR: 0.5161, 95% CI: 0.2874 to 0.912, Fig. 3d). Both male and female patients had a similar vascular involvement when they lager than 50 years old, with incidence ratios of 20% (21/105) and 23.53% (44/187) respectively.

### Aneurysm and pseudoaneurysm

Of all the 923 BD cases studied, 17 (1.84%) cases had aneurysm or pseudoaneurysm as vascular involvement. There were 13 cases in male patients and four in the females (*p* < 0.05, OR: 3.221, 95% CI: 1.097 to 9.112). Sixteen patients were less than 50 years old, while only one male patient had an aneurysm in his 70s. It appeared that most aneurysms and pseudoaneurysms attacked younger BD patients. However, because of the small number cases, there was no statistical significance (Table 1).

**Table 1:** Comparisons of aneurysm or pseudoaneurysm in BD patients with different age and gender

Comparison contents	Grouping	Aneurysm or pseudoaneurysm	BD without aneurysm	Chi-square (and Fisher’s exact) test
Age	< 50 y/o	16	720	*P* > 0.05, OR: 4.133, 95% CI: 0.7136 to 43.81
	≥ 50 y/o	1	186	
Gender	male	13	455	*P* < 0.05, OR: 3.221, 95% CI: 1.097 to 9.112
	female	4	451	

### Development of vascular involvement

**Fig. 4:**
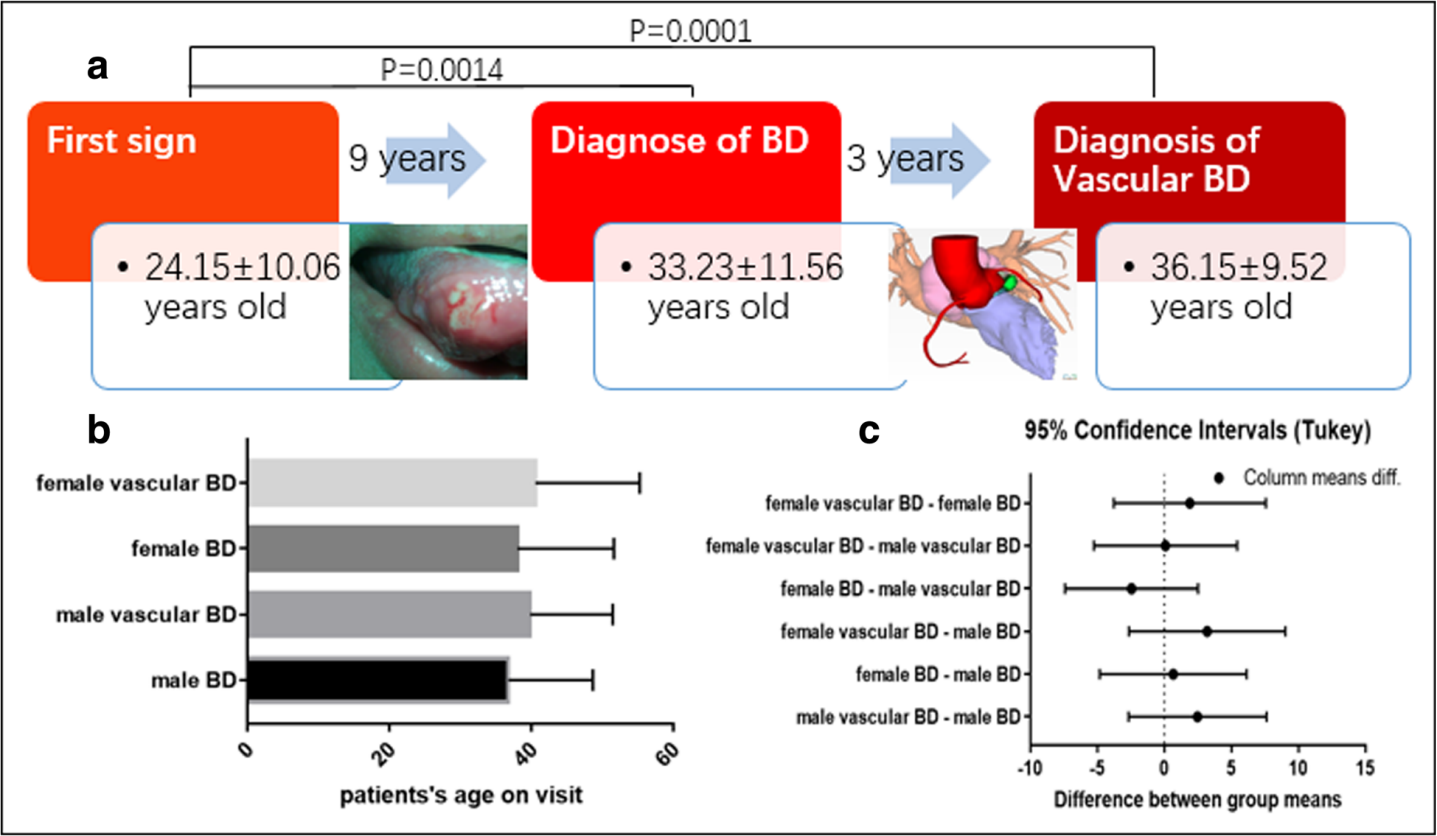
**a** By retrospect 20 patients’ history of progression, their diagnosis age and onset of vascular involvement age were higher than the first sign of BD with statistical significance (*p* = 0.0014 and 0.0001 respectively). It seems needs longer time to develop of vascular involvement, however, there is no statistical difference between BD diagnosis age and BD with vascular involvement. **b** & **c** Both in male and female patients, mucocutaneous BD and vascular BD showed no statistical age differences

As the most common seen first sign of BD is oral ulceration, patients are usually diagnosed with BD when genital ulceration, erythema nodosum, or uveitis appear a few years later. We speculated there is an interval to development of vascular lesions in BD. The medical history did not provide the detailed information we needed. Therefore, we invited 20 patients with vascular involvement patients to follow-up with us. We inquired when they had first sign, when they were diagnosed with BD and when was the vascular involvement detected. We found that the interval time between patients’ first manifestation to fulfilling the diagnostic criteria for BD were approximately 9 years, and an average of 12 years interval were required to involve the vascular system from first sign. Nevertheless, there was no statistical significance between the age of BD diagnosis and vascular system involvement (Fig. 4a). In fact, some patients manifested with vascular symptoms as the presenting sign in clinic or combined with other signs including oral ulceration.

Our database provided patient age at the visit, but did not reveal the age when BD or vascular BD was diagnosed. However, when comparing patients with mucocutaneous (*n* = 143) and vascular BD, we found that both in male and female patients, mucocutaneous BD and vascular BD showed no statistical age differences (Fig. 4b and c).

## Discussion

Comparatively, BD is not very common seen disease in clinic with an estimated prevalence rate ranges from 13.5 to 20 cases per 100,000 in Japan, Korea, China, Iran, and Saudi Arabia, whereas it is much lower in Western countries: 0.64 per 100,000 in the United Kingdom and 0.12 to 0.33 per 100,000 in the United States [6]. As vascular involvement takes up of 5–30% as reported [3], it has not been investigated in large samples. Koç Y et al. reported 137 Japanese patients with BD, of whom 38 had vascular involvement with a prevalence of 27.7%; the male to female ratio was 4.4 [7]. Tohmé et al. reported a prevalence of 12.9% in Lebanon, and men with BD were more likely to have vascular involvement (13/77, 17%) than were women (5/63, 8%) (*P* = 0.12) [8]. Our team carefully reviewed 923 BD candidates with accurate medical histories. All patients had appropriate radiography screening or confirmation. We found that 166 (17.98%) BD patients had vascular involvement. Results from our institution were in accordance with those of previous studies in that males were more susceptible to vascular involvement than were females. (OR: 1.868, *p* = 0.0004, 95% CI: 1.317 to 2.625).

As in our previous report, intestinal BD patients had a higher ESR and CRP levels than did mucocutaneous BD patients [9]. We incidentally found that CRP in intestinal BD patients was substantially higher than in vascular BD patients (*p* < 0.0001). CRP is an acute-phase protein of hepatic origin that increases following interleukin-6 secretion by macrophages and T cells, reflecting level of inflammation. This indicates the increased CRP more commonly seen in acute or active inflammatory reactions in intestinal BD patients. Ridker et al. [10] reported CRP was a significant predictor of the risk of cardiovascular events in women. Our study did not have a healthy control group, this is the limitation of this study. However, vascular BD appeared to be higher than mucocutaneous BD did, although the current date did not show statistical difference. Intestinal BD is not the topic of this study. We will give more attention in this point in future studies.

A portion of patients had vascular disease at more than one site, and in both male and female patients, the most common involved vessels were intracranial vessels and lower limb vessels. All ages of BD patients might present with vascular involvement. Of 166 vascular patients, 73.49% (122/166) patients were less than 50 years old and 26.51% (44/166) were equal to or greater than 50 years old. The incidence of vascular involvement in younger and older patients were similar, with ratios of 16.58% (122/736) and 23.53% (44/187), respectively. One interesting phenomenon in our study was that in premenopausal patients, the prevalence of vascular involvement was lower than that of postmenopausal patients (11.43% (40/350) vs. 20% (21/105), *p* = 0.0328, OR: 0.5161, 95% CI: 0.2874 to 0.912). The postmenopausal prevalence rate was similar to that of males. This trend was similar with primary cardiovascular disease in women [11]. Therefore, we believe gender or hormones in female BD patients might protect from vascular involvement. According to current studies, both secondary vascular disease in BD and primary vascular disease usually share the same pathogenesis. For example, a high level of homocysteine in the blood (hyperhomocysteinemia) makes a person more prone to endothelial cell injury, leading to inflammation in the blood vessels. This in turn may lead to atherogenesis, resulting in ischemic injury. Hyperhomocysteinemia has been correlated with the occurrence of blood clots, heart attacks and strokes [12]. Ramazan et al. [13] reported flow-mediated dilation in patients with BD was smaller than that of health subjects, and mean plasma homocysteine levels in patients with BD were significantly higher. On regression analysis, mean plasma homocysteine concentration was independently related to flow-mediated dilation, indicating that homocysteine-promoting oxidative stress was perhaps one of the mechanisms responsible for vascular injury in BD. Much evidence suggested hyperhomocysteinemia may be considered to be associated with thrombosis in BD patients [14]. It has been reported that high levels of homocysteine were diminished by estrogen [15]. Furthermore, estrogen replacement therapy for atherosclerosis has been employed for a long time [16]. These studies on primary vascular disease also inspired us regarding vascular BD treatment.

Aneurysms or pseudoaneurysms are lethal types of vascular BD. Of all 923 BD cases studied, 17 had aneurysms or pseudoaneurysms (1.84%). Male patients were more susceptible to aneurysms or pseudoaneurysms than were females (*p* < 0.05, OR: 3.221, 95% CI: 1.097 to 9.112). Although there was no a significant difference, it appeared that most aneurysms or pseudoaneurysms were diagnosed in younger BD patients. This may be because aneurysms or pseudoaneurysms BD patients may have shorter life spans. We will follow-up with these patients and provide more accurate epidemiology data.

We assumed that vascular impairment might be caused by inflammatory reaction of vessels in BD patients who were not well managed, and that a certain duration is required for mucocutaneous BD to progress into vascular BD. This seems to be a reasonable speculation. However, by following-up with 20 vascular BD patients revealed that there is no significant age difference between the two time points. We also analyzed the age of all patients on their visit to our institution, and there were no differences between mucocutaneous BD and vascular BD patients in both males and females. Although limited by the medical record, patients’ age on visiting might be actually older than when they were diagnosed. But since both vascular and mucocutanous BD cases were enrolled in this cross sectional study without selection, so this raw observation could eliminate the bias and reflect that there is no time episode from mucocutanous type to vascular involvement.

## Conclusions

In conclusion, from our retrospective study with large numbers of samples collected, we established a demography of vascular BD at different genders and ages. In accordance with the literature from other countries, we found that male BD was susceptible to progression to vascular involvement. The most commonly involved areas were cerebral and lower limb vessels. Some novel findings were obtained from our study: the observation that postmenopausal BD patients had a similar incidence as did males; aneurysms or pseudoaneurysms were more common in male BD patients; and there were no evidence of patients in our study with established BD to develop vascular manifestations.

## Methods

### Patients

From October 2012 to October 2017, 1127 BD patients visited our clinic or were admitted to the Rheumatology Department at Huadong Hospital. All patients met the diagnostic criteria of the International Study Group for Behçet’s Disease ([17, 18]). We excluded patients with cancer, rheumatoid arthritis, diabetes, syphilis, HIV, and those without full-body check-up (including skull MRI, chest CT, gastrointestinal endoscopy, and B-US echocardiography). Patients with latent infections with Epstein-Barr virus, hepatitis virus, *mycobacterium tuberculosis*, and herpes simplex virus were included, as these conditions were considered risk factors for BD [19]. In all, 923 BD patients from 36 provinces in China were enrolled.

There are no generally-accepted diagnostic criteria for vascular BD, and it is difficult to distinguish vascular lesions caused by BD from primary vasculopathies. Considering the similar pathogenesis shared by primary and secondary atherosclerosis, venous thrombosis, and aneurysms, ([14, 19]) all patients with vascular disease/symptoms manifesting after BD manifestations were diagnosed with vascular BD.

### Data collection

We collected the following patient information: gender; age; clinical manifestations: age of onset, initial symptoms, duration of disease, clinical signs, and evidence of systemic impairment; and blood examinations. Data were stored in MS-Excel for further analysis. All participants underwent B-ultrosound for cardiovascular assessment including vessels in the neck and the four extremities, as well Magnetic Resonance Imaging (MRI) for skull involvement. For patients with chest or abdominal pain or history of vascular involvement, computer tomography (CT) scan for aorta, and their branches were recommended. A rheumatologist confirmed diagnosis of BD. BD with different organs involvement were further confirmed by consultations with specialists of their specialty (i.e., ophthalmologist, radiologist, vascular surgeon and gastroenterologist).

### Statistical analysis

Statistical was performed using GraphPad Prism 7.0 software. The incidence rates of vascular BD and BD with aneurysm or pseudoaneurysm at various ages (< 50 years old and ≥ 50 years old) and genders were analyzed by Chi-square (and Fisher’s exact) test. The erythrocyte sedimentation rate (ESR), C-reactive protein (CRP), and age of various types of BD (mucocutaneous, intestinal and vascular) were analyzed by ordinary one-way ANOVA test. *P* < 0.05 was considered statistically significant.

## Abbreviations

95%CI: 95% confidence interval
BD: Behcet’s disease
CRP: C-reactive protein
ESR: Erythrocyte sedimentation rate
OR: Odds ratio
